# Targeted parallel DNA sequencing detects circulating tumor‐associated variants of the mitochondrial and nuclear genomes in patients with neuroblastoma

**DOI:** 10.1002/cnr2.1687

**Published:** 2022-07-28

**Authors:** Lara Riehl, Medhanie Mulaw, Katharina Kneer, Meinhard Beer, Ambros Beer, Thomas F. Barth, Vladimir Benes, Johannes Schulte, Matthias Fischer, Klaus‐Michael Debatin, Christian Beltinger

**Affiliations:** ^1^ Department of Pediatrics and Adolescent Medicine University Medical Center Ulm Ulm Germany; ^2^ Institute of Experimental Cancer Research University Medical Center Ulm Ulm Germany; ^3^ Department of Nuclear Medicine University Medical Center Ulm Ulm Germany; ^4^ Department of Diagnostic and Interventional Radiology University Medical Center Ulm Ulm Germany; ^5^ Department of Pathology University Medical Center Ulm Ulm Germany; ^6^ Genomics Core Facility European Molecular Biology Laboratory (EMBL) Heidelberg Germany; ^7^ Pediatric Oncology and Hematology Charité University Medicine Berlin Germany; ^8^ German Cancer Research Center (DKFZ) German Cancer Consortium (DKTK) Heidelberg Germany; ^9^ Department of Pediatric Oncology and Hematology University Children's Hospital of Cologne Cologne Germany

**Keywords:** liquid biopsy, mitochondrial and nuclear genomes, mutational signatures, neuroblastoma

## Abstract

**Background:**

The utility for liquid biopsy of tumor‐associated circulating single‐nucleotide variants, as opposed to mutations, of the mitochondrial (mt) and nuclear genomes in neuroblastoma (NB) is unknown.

**Procedure:**

Variants of the mt and nuclear genomes from tumor, blood cells, and consecutive plasma samples of five patients with metastatic NB that relapsed or progressed were analyzed. Targeted parallel sequencing results of the mt genome, and of the coding region of 139 nuclear genes and 22 miRNAs implicated in NB, were correlated with clinical imaging and laboratory data.

**Results:**

All tumors harbored multiple somatic mt and nuclear single nucleotide variants with low allelic frequency, most of them not detected in the circulation. In one patient a tumor‐associated mt somatic variant was detected in the plasma before and during progressive disease. In a second patient a circulating nuclear tumor‐associated DNA variant heralded clinical relapse. In all patients somatic mt and nuclear variants not evident in the tumor biopsy at time of diagnosis were found circulating at varying timepoints. This suggests either tumor heterogeneity, evolution of tumor variants or a confounding contribution of normal tissues to somatic variants in patient plasma. The number and allelic frequency of the circulating variants did not reflect the clinical course of the tumors. Mutational signatures of mt and nuclear somatic variants differed. They varied between patients and were detected in the circulation without mirroring the patients' course.

**Conclusions:**

In this limited cohort of NB patients clinically informative tumor‐associated mt and nuclear circulating variants were detected by targeted parallel sequencing in a minority of patients.

## 1 INTRODUCTION

Analyses in patients with various tumors have shown promising results using circulating cell‐free cancer‐associated DNA mutations for liquid biopsy.^1^, ^2^, ^3^ While circulating mutations from the nuclear cancer genome have been investigated intensively in liquid biopsies, little is known about the mitochondrial (mt) genome for this purpose. The mt genome consists of 16 569 bp.^4^ Since up to 10 mt genome copies are found within one mitochondrion and up to 1000 mitochondria are present per cell, each cell may harbor up to 1 × 10^4^ copies of the mt genome.^5^ The mutational rate in mitochondria is thought to be high due to production of reactive oxygen species in the respiratory chain, lack of protective histones and insufficient repair.^6^ Mt mutations and variants are present in many cancers.^7^ There are emerging clues that tumor‐associated mt genome variants may be detectable in circulating tumor DNA.^8^

A variant base together with the bases immediately 3′ and 5′ of the variant form a so‐called motif. A motif provides additional, contextual information about the variant. The six possible classes of base mutations can form 96 possible motifs. By calculating the frequencies of such mutational motifs a mutational signature is obtained.^9^ The number of variant motifs is principally higher than the number of single‐base variants, thus increasing the number of both mt and nuclear and variants potentially informative for liquid biopsy. This may be particularly helpful in neuroblastoma (NB), where the mutational load to be low. However, it is unknown whether circulating mt or nuclear tumor‐associated signatures are useful as biomarkers in liquid biopsy of tumors in general and of NB in particular.

Molecularly, NB is characterized mainly by changes in copy number alterations, many of which carry a poor prognosis and are used to stratify patients into high‐risk groups. Prominent among these are amplification of *MYCN* and segmental chromosome alterations, which can be detected by liquid biopsy in patients with NB.^10^, ^11^, ^12^, ^13^, ^14^, ^15^, ^16^, ^17^ Copy number profiling of the whole NB genome by liquid biopsy is feasible.^14^, ^18^, ^19^

In contrast to copy number alterations, recurrent mutations are rare in NB. These include mutations of *ALK*, *ATRX*, *LMO1*, genes of the RAS‐RAF‐MAPK pathway, as well as genomic rearrangements of *TERT*.^20^, ^21^, ^22^, ^23^, ^24^, ^25^, ^26^, ^27^, ^28^, ^29^, ^30^, ^31^, ^32^ Given the paucity of recurrent mutations in NB, few investigations have addressed the utility of such mutations as tumor‐associated circulating biomarkers in NB. Along this line, the mutational status of ALK at its F1174 and R1275 mutational hotspots has been reliably determined in peripheral blood of NB patients.^33^ Little is known about the utility for liquid biopsy of tumor‐associated circulating single nucleotide variants, as opposed to mutations, obtained from limited targeted panel sequencing.

Many NB harbor tumor‐associated mt variants^34^, ^35^ and relapsed NB display enhanced evolutional changes and spatio‐temporal differences of tumor‐associated mt variants.^34^ It is yet unknown, whether tumor‐associated mt variants can be determined in liquid biopsy of NB.

In addition to tumor DNA, microRNAs have been detected in the blood of mice and NB patients, thus constituting potential circulating tumor‐associated biomarkers for NB.^36^ Neuroblastoma‐derived exosomes containing proteins involved in tumor progression (PROG)^37^ may also be possible circulating biomarkers. Other mRNA and protein candidates for NB biomarkers may be found among the predictive 42‐gene classifier developed by integrating eight independent studies.^38^ Recently, an expression signature of five NB genes in blood of patients with relapsed/refractory NB was shown to correlate with PROG‐free survival and to improve definition of disease status.^39^

In a small cohort of patients with progressing or relapsing NB we detected informative circulating tumor‐associated mt and nuclear variants by targeted sequencing in a minority of patients.

## 2 METHODS

### 2.1 Overview

**FIGURE 1:**
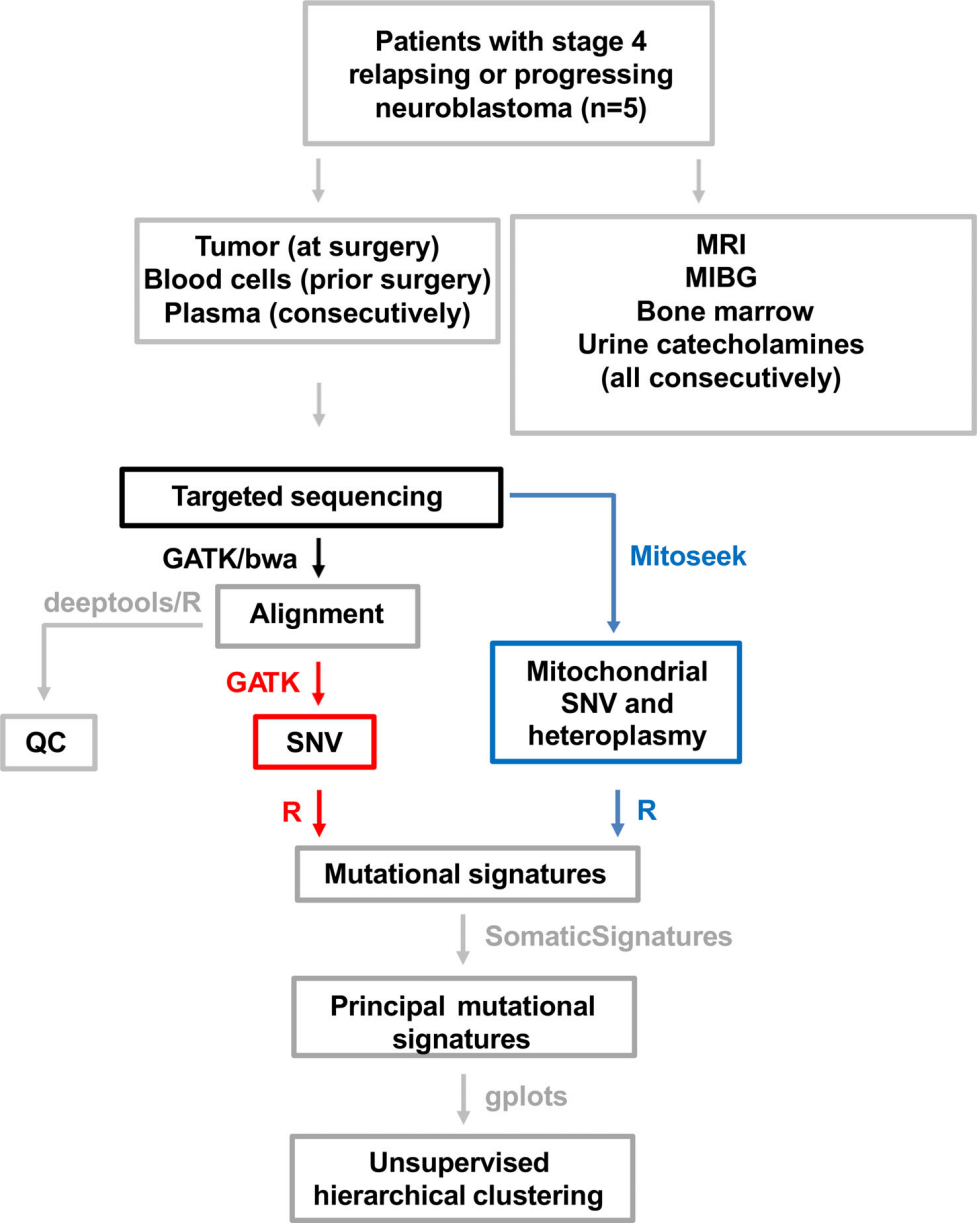
Study overview. The disease course of patients with stage 4 relapsing or progressing NB was studied. DNA from tumor, blood cells (germline), and consecutive plasma samples of the patients were subjected to targeted parallel sequencing. For comparison, tumor burden, as determined by MRI and MIBG scans, urine catecholamine metabolites and bone marrow biopsies were assessed consecutively. After targeted sequencing raw reads were aligned to human reference genome using *bwa* and *GATK* tools. Quality control (QC) analyses including alignment efficiency, and target region coverage (depth and extent of coverage) were performed using *deeptools* and custom *R* scripts. *GATK* and *cnvkit* were used for nuclear SNV analysis. Mt read extraction and alignment, mt SNV calling and heteroplasmy analysis were performed using *MitoSeek*. SNV‐based somatic mutational signatures analysis were determined using *R*. Principal mutational signatures were derived using the *Bioconductor* package *SomaticSignatures*. Unsupervised hierarchical clustering and heatmapping of principal signatures were performed using the Bioconductor package *gplots*.

Figure 1 provides an overview of patients, disease assessment, targeted sequencing, and bioinformatic analysis of this study. In this article, we adhere to the following definitions. Any single nucleotide alteration is called a variant. Variants are considered germline when detected in peripheral blood cells, and somatic when they are not. Somatic variants detected in tumor samples are called tumor‐associated variants. Variants detected in patient plasma, which is cell‐free, are called circulating variants.

### 2.2 Patients

All patients with NB treated at the Department of Pediatrics and Adolescent Medicine of the University Medical Center Ulm between October 2012 and March 2018, and from whom sufficient tumor DNA, germline DNA and consecutive plasma samples were available in adequate quality, were included in the investigation. The study was approved by the local ethics committee and informed consent was obtained. A total of five patients were chosen based on having stage 4 disease, reasoning that initial metastatic disease facilitates detection of circulating variants. One patient went into complete remission (CR) during monitoring and then relapsed, while four showed partial remission (PR) and then progressed. A detailed description of the patients is given in Table S1.

### 2.3 Disease assessment

Vanillylmandelic acid and homovanillic acid in spot urine, and bone marrow cytology and histology were analyzed by standard methods.

Tumors were imaged by magnetic resonance imaging using a 3T device (Magnetom Skyra MRI, Siemens Healthineers, Erlangen, Germany). MIBG scanning was performed by a dual‐head gamma camera (Symbia TruePoint SPECT/CT, Siemens Healthineers) 4 and 24 h after intravenous application of Iodine‐123‐MIBG (Metaiodobenzyl‐guanidin). In sum, 77 MRIs and 2 CTs of the whole body or abdomen as well as 33 MIBG scans were evaluated.

The mintLesion program (Version 3.2.1, Mint Medical) was used to calculate tumor burden at each time point. Briefly, tumor volume of primary tumor and metastases in lymphnodes, liver, lung, brain, and peritoneal cavity was determined. The volume of bone marrow metastases was added. The volume of bone marrow metastases was calculated incorporating related approaches form other imaging methods.^40^, ^41^, ^42^ First, a reference was created for the total volume of bone marrow present in children in general using magnetic resonance data of three representative patients of different age (3 months, 1 year, 4 years). Then, patients' MIBG scintigraphy scans were evaluated for their MIBG‐distribution in bone marrow, and the distribution was set in correlation to the established reference to calculate the metastatic volume affecting the total bone marrow. Thus, the total tumor burden of each patient consisted of the volumetric sum defined by MRI‐ and MIBG‐scans.

### 2.4 Sample procurement and assessment

Tumor samples were obtained at the time of surgery. DNA samples from the NB were either provided by the Tumorbank Embryonale Tumoren, Unikinderklinik Köln, Cologne, Germany or isolated from formalin‐fixed paraffin‐embedded tumor tissue slides provided by the Department of Pathology, University Medical Center Ulm, Germany. DNA was isolated using the GeneJet FFPE DNA Purification Kit (ThermoScientific, Waltham, Miami, USA) from three or four slides per patient measuring 8 mm in depth.

Germline DNA of each patient was extracted from mononuclear blood cells after diagnosis with a time lag to surgery, using the QIAamp DNA Blood Mini Kit (Qiagen, Venlo, Netherlands).

From each patient, consecutive peripheral blood samples were collected into EDTA tubes and processed within 1.5 h. Depending on the size of the child, 1.6 to 4.9 ml blood was collected. Samples were centrifuged at 800*g* for 10 min at 4°C. The plasma was subjected to a second round of centrifugation at 14.000 rpm for 10 min at 4°C. The supernatant was aliquoted and stored at −80°C. Ct DNA was isolated from one aliquot of plasma with the QIAamp Circulating Nucleic Acid Kit (Qiagen, Venlo, Netherlands) following the manufacturer's instructions. Following elution with 30 μl a second elution step was performed with 20 μl, resulting in a total volume of 50 μl per sample.

### 2.5 Design of a targeted parallel sequencing panel for NB

A custom DNA targeted parallel sequencing panel for NB was designed using Agilent's SureDesign® online software tool. Besides the whole mt genome the panel encompassed the coding regions of nuclear genes selected from three sources. First, genes implicated in NB by literature,^25^, ^26^, ^27^, ^28^ second, 25 nuclear genes and 22 miRNAs recurrently mutated in at least five patients in the cohort of the TARGET NB consortium (http://target.nci.nih.gov/dataMatrix/), and finally genes not implicated in NB so far but deemed to be potentially relevant based on their function. The 139 nuclear genes and 22 miRNAs are listed in Table S2. In addition, regions of interest randomly scattered within the most common structural variations in NB located in chromosomal regions 1q, 1p, 3p, 11q, and 17q, as well as the *ATRX* and *TERT* loci, were included in the panel (Table S3).

The regions of interest were designed to cover each base one time or, for problematic regions previously identified, three times. The complete panel comprised 48 341 probes covering 2.319 Mb.

### 2.6 Targeted parallel sequencing

Unique patient‐specific libraries from cell‐free plasma samples, and from tumor cells and blood cells (germline) were prepared utilizing ThruPLEX Plasma‐Seq or ThruPLEX Tag‐Seq kits (Rubicon Genomics, Ann Arbor, MI, USA), respectively. Libraries were prepared according to the manufacturer's protocol. 6–18 ng DNA from cell‐free plasma samples, and 5–50 ng DNA from tumor and blood cells were used. Each library was amplified with 10 cycles and assigned an individual index. A unique molecular tag was added to identify PCR duplicates.

Target regions were enriched using the SureSelectXT kit with the custom capture panel. Hybridization and capture were performed according to the manufacturer's protocol, with modifications. IDT xGen universal blocking oligonucleotides (Integrated DNA Technologies, Leuven, Belgium) were added to the SureSelect Block Mix before hybridization. The postcapture target amplification step was modified by omitting the indices and switching to P5 and P7 primers (Illumina, San Diego, CA, USA) instead of the indexing postcapture primer. The optimal cycle number for target amplification was determined to be 12, as assessed by the KAPA library quantification kit (Roche, Basel, Switzerland). DNA concentration and average fragment size were measured by Tape Station (Agilent, Santa Clara, CA, USA), and libraries were pooled equimolarily to 4 nM. Sequencing was performed on a NextSeq 500 device (Ilumina) with 75 PE and high‐output kit mode.

### 2.7 Bioinformatic and statistical analysis

An in‐house bioinformatics pipeline was used for sequence analysis and annotation following *GATK* guidelines.^43^ Briefly, raw fastq files were mapped to the human reference genome (Genome Reference Consortium human genome build 37, human genome 19) after quality control and adapter trimming. Base calls differing from the reference sequence and passing filtering criteria were identified as variants. These variants were first compared with dbSNP (http://www.ncbi.nlm.nih.gov/projects/SNP/index.html) and 1000 genomes (http://www.1000genomes.org/). Duplicate removal was performed via *Picard* (http://broadinstitute.github.io/picard/). Somatic nuclear variants or mutations were filtered using *Mutect2* (https://gatk.broadinstitute.org/hc/en-us/articles/360037593851-Mutect2) by comparing to the germline control sample. *MitoSeek* version 1.3^44^ was used to map mt sequencing reads to the revised Cambridge reference sequence, to call mt somatic variants and to determine heteroplasmy. mt variants were classified using the Human Gene Mutation Database (http://www.biobase-international.com/product/hgmd). Individual signatures were generated using *R* and subjected to principal component analysis as a statistical approach to capture the main sources of variation in each patient, which are henceforth called principal signatures. Principal component analysis was done using the *Bioconductor* package *SomaticSignatures*.^45^ Unsupervised hierarchical clustering was performed as an unbiased statistical analysis to assess if a principal signature was associated with the clinical course of the patients. Unsupervised hierarchical clustering and heatmapping of principal signatures were conducted using the *Bioconductor* package *gplots*.^46^

## 3 RESULTS

### 3.1 Targeted parallel sequencing

Mean depth coverage of the targeted nuclear regions was 296× in blood cells (germ line), 117× in tumor samples and 169× in plasma samples. Mean depth of coverage of the mt genome was 7774× in blood cells, 7827× in tumor samples, and 990× in plasma samples.

### 3.2 Tumor‐associated variants and circulating somatic variants are present in all patients

**FIGURE 2:**
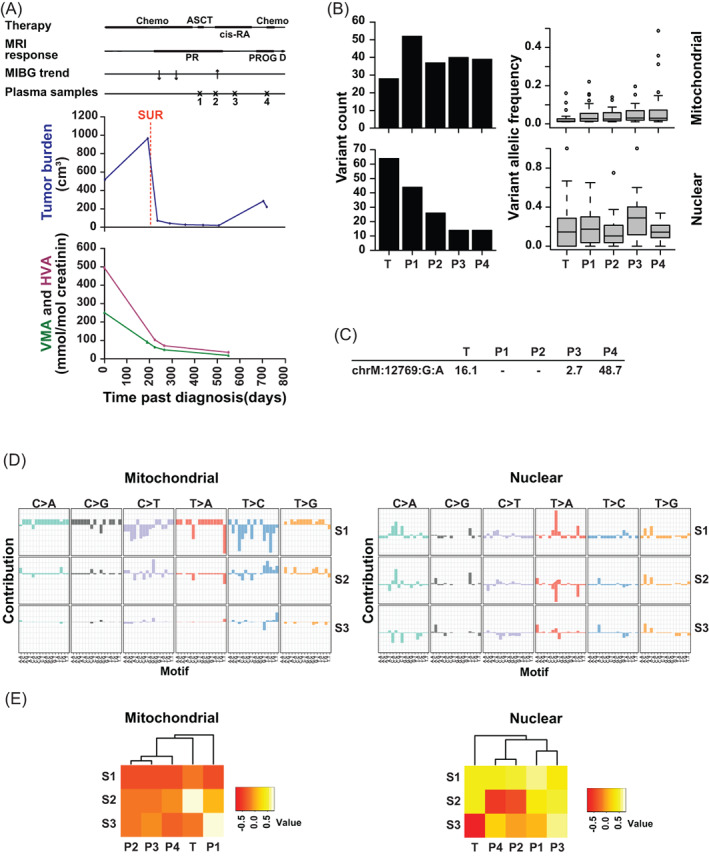
A circulating tumor‐associated mt variant in a patient before and during progression. (A) Clinical course. The upper panel shows the time lines post diagnosis of therapy (Chemo, chemotherapy; ASCT, autologous stem cell transplantation; cis‐RA, cis‐retinoic acid differentiation therapy), of response assessment by MRI (PR, partial response; PROG, progression), of death (D), of response assessment by MIBG and of plasma sampling. The middle panel depicts tumor burden of primary tumor and metastases, as determined by MRI and MIBG scans, and time of tumor surgery and biopsy (SUR). The lower panel shows urine levels of vanillylmandelic acid (VMA) and homovanillic acid (HVA). (B) Numerous mt and nuclear somatic variants detected at low allelic frequency in tumor and consecutive plasma samples. Shown is the number of mt and nuclear variants (left panels) and the frequency of variant alleles (right panels; mt variant allele frequency corresponds to heteroplasmy) in tumor and at consecutive times of plasma sampling. T denotes tumor sample at initial diagnosis and B1–B4 denote plasma samples at consecutive time points. (C) Appearance of a circulating tumor‐associated mt variant at time of disease progression. The variant, its occurrence and its allelic frequency are depicted. (D) Tumor‐associated mt and nuclear principal signatures differ. The top three signatures derived from principal component analysis and designated “principal signatures” S1, S2, and S3 are depicted. The base substitutions of the motifs are shown above each plot while the flanking bases are shown below the plots. (E) Circulating mt and nuclear principal signatures do not reflect the clinical course. Unsupervised hierarchical clustering of the mitochondrial and nuclear principal signatures S1, S2, and S3 by the consecutive samples T (tumor) and P1–4 (plasma samples) are shown.

**FIGURE 3:**
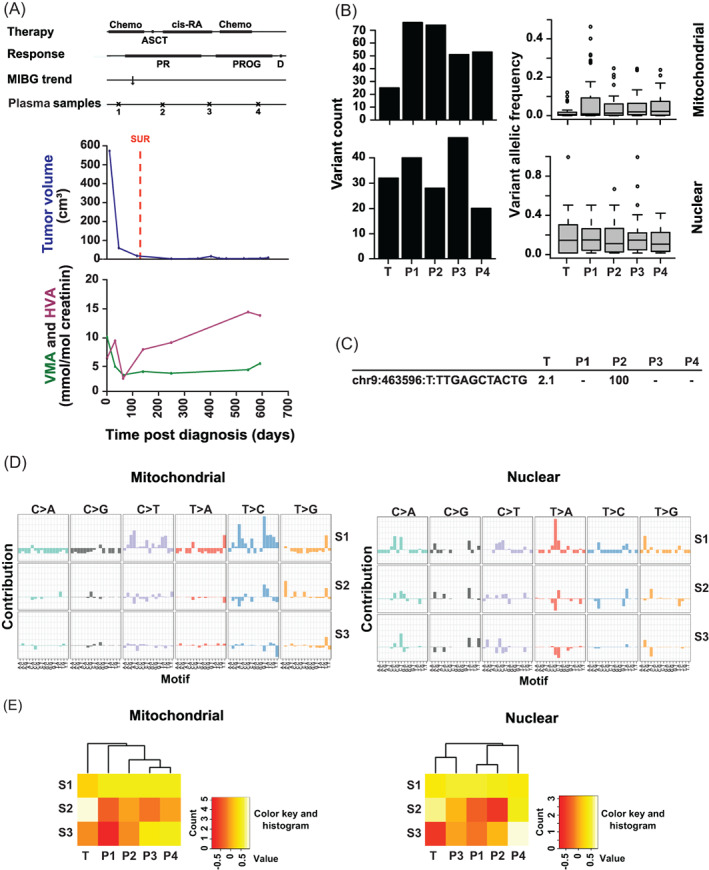
Appearance of a circulating tumor‐associated nuclear variant in a patient before progression. (A) Clinical course. Chemo, chemotherapy; ASCT, autologous stem cell transplantation; cis‐RA, cis‐retinoic acid differentiation therapy; PR, partial remission; PROG, progression; D, death; MIBG trend, response assessment by MIBG; Plasma samples, time of plasma sampling; SUR, surgery and biopsy; VMA, vanillylmandelic acid; HVA, homovanillic acid. (B) Numerous nuclear and mt somatic variants with low allelic frequency in tumor and consecutive plasma samples. T, tumor; P1–P4, consecutive plasma samples. (C) A circulating tumor‐associated nuclear variant occurring before progression. The variant, its occurrence and its allelic frequency are depicted. T, tumor; P1–P4, consecutive plasma samples. (D) Mitochondrial and nuclear principal signatures differ. Mt and nuclear principal signatures S1, S2, and S3 are shown. (E) Circulating mt and nuclear principal signatures do not reflect the clinical course. Unsupervised hierarchical clustering of the principal signatures S1, S2, and S3 by the consecutive samples T (tumor) and P1–4 (plasma samples) are depicted.

**FIGURE 4:**
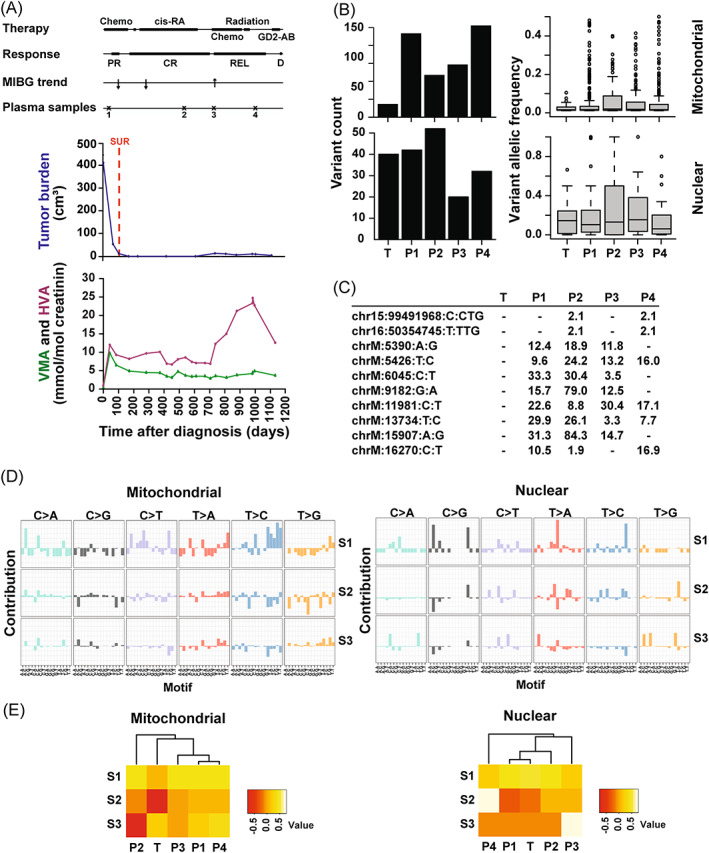
Multiple circulating somatic mt variants in a patient during complete remission and subsequent progression. (A) Clinical course. Chemo, chemotherapy; cis‐RA, cis‐retinoic acid differentiation therapy; radiation, radiotherapy; GD‐2 AB, anti‐GD2 antibody therapy; PR, partial remission; CR, complete remission; REL, relapse; D, death; MIBG trend, response assessment by MIBG; Plasma samples, time of plasma sampling; SUR, surgery and biopsy; VMA, vanillylmandelic acid; HVA, homovanillic acid. (B) Numerous mt and nuclear somatic variants with low allelic frequency in tumor and consecutive plasma samples. T, tumor; P1–4, consecutive plasma samples. (C) Many circulating mt and nuclear somatic variants in consecutive plasma samples not seen in the tumor sample. The variant, its occurrence and its allelic frequency are depicted. T, tumor; P1–P4, consecutive plasma samples. (D) Mt and nuclear principal signatures differ. Mt and nuclear principal signatures S1, S2, and S3 are shown. (E) Circulating mt and nuclear principal signatures do not reflect the clinical course. Unsupervised hierarchical clustering of the principal signatures S1, S2, and S3 by the consecutive samples T (tumor) and P1–4 (plasma samples) are depicted. [Correction added on 08 August 2022, after first online publication: The bottom graph of Figure 4A has been corrected in this version].

**FIGURE 5:**
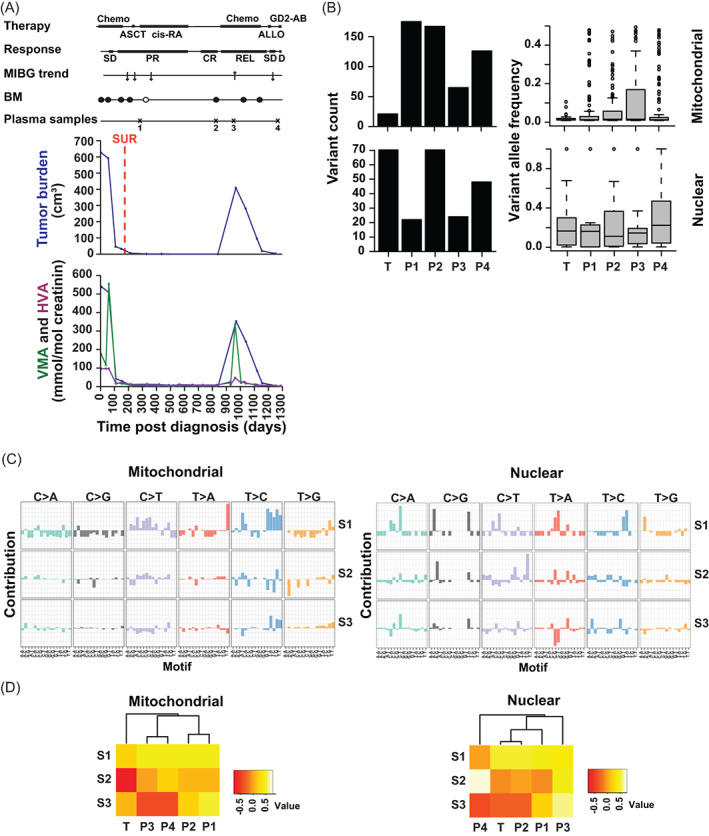
No circulating tumor‐associated variants in a patient with lethal disease. (A) Clinical course. Chemo, chemotherapy; ASCT, autologous stem cell transplantation; cis‐RA, cis‐retinoic acid differentiation therapy; ALLO, allogeneic stem cell transplantation; GD‐2 AB, anti‐GD2 antibody therapy; SD, stable disease; PR, complete remission; CR, complete remission; REL, relapse; D, death; MIBG trend, response assessment by MIBG; BM, bone marrow samples (empty circle: negative for NB cells, filled circles: positive for NB cells); Plasma samples, time of plasma sampling; SUR, surgery and biopsy; VMA, vanillylmandelic acid; HVA, homovanillic acid. (B) Many mt and nuclear somatic variants with low allelic frequency in tumor and consecutive plasma samples. T, tumor; P1–4, consecutive plasma samples. (C) Mt and nuclear principal signatures differ. Mt and nuclear principal signatures S1, S2, and S3 are depicted. (D) Circulating mt and nuclear principal signatures do not reflect the clinical course. Unsupervised hierarchical clustering of the principal signatures S1, S2, and S3 by the consecutive samples T (tumor) and P1–4 (plasma samples) are shown.

Tumor‐associated mt variants were found in all tumors, with their number and allelic frequency ranging from 21 to 40 (median 29), and 1.1% to 49.3%, respectively. Tumor‐associated somatic nuclear variants were also present in all tumors. Their number and allelic frequency ranged from 32 to 70 (median 58), and 1.1% to 90%, respectively (Figures 2, 3, 4, 5 and Figure S1).

In two patients tumor‐associated variants were also detected in the patients' plasma (Table S4). In all patients somatic mt and nuclear variants not evident in the tumor biopsy at time of diagnosis were found to circulate at varying timepoints (Table S4).

### 3.3 Occurrence of a circulating tumor‐associated mt variant in a patient before and during progressive disease

This patient presented with a *MYCN*‐amplified, undifferentiated NB of the right adrenal gland metastasizing to distant lymph nodes and liver (Patient A, Table S1). After PR the disease progressed and the patient died (Figure 2A). More nuclear than mt somatic variants were seen in the tumor (Figure 2B). The allelic frequency of most variants was low, in particular of the mt variants, which were all heteroplasmic. A substantial number of both mt and nuclear somatic variants were detected in consecutive plasma samples. The number of variants in the plasma samples, as compared with the tumor sample, was higher for the mt variants (Figure 2B). At all time points of plasma sampling the number of mt variants was higher than the nuclear variants. Neither counts nor allelic frequencies of the variants changed in the plasma samples when the disease progressed. Of note, one of the tumor‐associated mt variants occurred in the plasma samples with increasing allelic frequency when the disease progressed (Figure 2C).

We investigated whether mt and nuclear signatures provided additional information in the liquid biopsies. Along this line, we generated principal signatures using principal component analysis to capture the primary sources of variation in mutational signatures. Indeed, most of the variation in the individual signatures could be condensed into three principal signatures, ranked and designated S1, S2, and S3 (Figure 2D). Principal signatures of the mt and nuclear tumor‐associated variants differed (Figure 2D). Unsupervised hierarchical clustering of the top mt and nuclear principal signatures S1, S2, and S3 by the tumor sample and the consecutive plasma samples did not reflect the clinical course of the patient (Figure 2E).

Taken together, one of the tumor‐associated mt variants was informative for liquid biopsy in this patient, while variant count, variant allele frequency, and principal mutational signatures were not.

### 3.4 Occurrence of a circulating tumor‐associated nuclear variant in a patient before progressive disease

The patient was diagnosed with a high‐risk, *MYCN*‐amplified, 1p‐deleted, undifferentiated NB of the right adrenal gland metastasizing to distant lymph nodes, bone and liver (Patient C, Table S1). After partial remission the disease progressed and the patient died of cerebral metastases (Figure 3A). Mt and nuclear somatic variants were present in the tumor (Figure 3B). The allelic frequency of most variants was low and all mt variants were heteroplasmic. Numerous somatic variants were detected in consecutive plasma samples. The number of variants in the plasma samples, as compared with the tumor sample, was higher for the mt variants (Figure 3B). At most points of plasma sampling the number of mt variants exceeded nuclear variants. Counts and allelic frequencies of the variants in the plasma samples were not altered during disease progression. Importantly, one of the tumor‐associated nuclear variants became evident in the plasma samples with a high allelic frequency before the disease progressed (Figure 3C), when it became undetectable. Principal mutational signatures of the mt and nuclear variants differed (Figure 3D) and did not reflect progression of the disease (Figure 3E).

In summary, a tumor‐associated nuclear variant was transiently informative in this patient, while variant count, variant allele frequency, and mutational signatures were not.

### 3.5 Multiple somatic mt variants in a patient during complete remission and subsequent progression

This patient was diagnosed with a NB originating from an adrenal gland that had metastasized to bone (Patient D, Table S1). The tumor was *MYCN*‐amplified and 1p‐deleted. The patient went into CR but relapsed at the initial site and eventually died of cerebral metastases (Figure 4A).

Compared with the tumor the number of somatic variants in the first plasma sample was higher for the mt but not for the nuclear variants (Figure 4B). At all time points of plasma sampling there were more mt than nuclear somatic tumor variants and most variants had low allelic frequencies. Variant counts and allelic frequencies did not change from CR to relapse (REL) and death. Interestingly, while no tumor‐associated variants were detectable in the circulation numerous somatic mt variations and two somatic nuclear variations not detected in the initial tumor sample were recurrently detected in the plasma samples (Figure 4C). A difference between mt and nuclear principal signatures was apparent (Figure 4D) while neither signature reflected REL and PROG of the disease (Figure 4E).

### 3.6 No circulating tumor‐associated variants in two patients with lethal disease

Patient B presented with a poorly differentiated NB of the right adrenal gland with metastases to bone marrow, distal lymphnodes and bone (Figure 5, Table S1). *MYCN* was not amplified and 1p was not lost. After remission the patient relapsed and received chemotherapy, haploidentical stem cell transplantation and ch14.18 antibody therapy (Figure 5A). The patient responded well but died shortly thereafter of encephalopathy of unclear etiology. Again, at all time points of plasma sampling there were more mt than nuclear somatic variants, most variants showed low allelic frequencies and both counts and frequencies did not reflect the clinical course (Figure 5B). Mt and nuclear and principal signatures differed (Figure 5C) while being uninformative about the clinical course (Figure 5D).

Patient E was diagnosed with an abdominal NB metastasizing to bone marrow and bone (Figure S1 and Table S1). *MYCN* copy number and 1p status were normal. The patient responded to therapy but then progressed and eventually succumbed to intracranial metastasis (Figure S1A). At most timepoints of plasma sampling mt somatic variants were more frequent and most variants had low allelic frequencies (Figure S1B). No tumor‐associated circulating variants were detected. Somatic circulating variant counts, allelic frequencies, and principal mutational signatures were uninformative regarding the patient's clinical course (Figure S1B‐D).

## 4 DISCUSSION

We investigated the utility for liquid biopsy of tumor‐associated circulating single nucleotide variants, as opposed to mutations, obtained by targeted sequencing using a panel covering the mt genome and NB‐relevant genes. Circulating tumor‐associated mt and nuclear variants were detected, demonstrating the potential of this approach for liquid biopsy of NB.

Mt variants contributed markedly to the overall number of tumor‐associated variants in this study. This occurred despite the much smaller mt genome compared with the nuclear genes that were sequenced. This reflects the very high copy number per cell of the mt genome, its higher mutational rate due to the high rate of radical production by the respiratory chain, and its increased vulnerability to DNA damage because of lack of histones and of other protective mechanisms.^47^ All tumor‐associated mt variants were heteroplasmic and had at low allelic frequency. This is consistent with the notion that these mt variants did not provide the NB cells with a survival advantage and that mt variants which impaired the function of the respiratory chain were selected against.

In one patient a circulating tumor‐associated mt variant followed the patient's disease course, suggesting it was informative. In all other patients, however, no circulating tumor‐associated mt variants were found. This could be explained by insufficient shedding of the majority of tumor‐associated mt variants from the tumor cells. In addition, the type and allelic frequency of the mt variants may have changed and decreased, respectively, during evolution of the tumor since diagnosis. Many somatic variants not seen in the diagnostic tumor sample were detected in the plasma samples. This may be accounted for by tumor heterogeneity in regard to tumor‐associated variants. Tumor‐associated mt variants only present in the part of the tumor not subjected to sequencing may have been shed into the circulation. In addition, recent studies have shown that variations of the mt genome are present in normal organs at low frequency and with organ specificity.^5^, ^48^, ^49^, ^50^ Thus, the somatic mt variants seen in the patients' circulation but not their tumor samples may not have been tumor‐derived. Supporting this notion, the number and allelic frequencies of circulating mt somatic variants did not reflect the clinical course of the patients.

Nuclear tumor‐associated variants were common. Their allelic frequency was low, in line with variants not being tumor drivers. A circulating nuclear tumor‐associated variant heralded PROG in one patient. However, in the majority of patients no circulating tumor‐associated nuclear variants were found while all had circulating somatic nuclear variants not seen in the diagnostic tumor sample and not reflecting the clinical course. Possible reasons for these findings are similar to those for mt somatic variants: insufficient shedding, tumor heterogeneity, and confounding shedding of nuclear variants from normal organs.^51^ In addition, the limited number of nuclear genes sequenced likely limited the number of circulating variants detected.

Mutational signatures could be determined in the somatic variants of the tumor samples and in the circulating somatic variants of the patients. We generated principal signatures by principal component analysis that identified a minimal set of significant individual circulating somatic variant signatures to serve as markers for disease status. The top mt principal signature S1 contains a prominent T>C transition with a specific subset of flanking bases. In addition, enrichment of the C>T transition was seen, with no preference for specific flanking bases. Both changes are in line with what others and we have shown for individual signatures of NB tumor samples and their relapses,^34^, ^52^ and constitute a replication‐dependent pattern. In contrast to the top mt principal signature S1, the top nuclear principal signature S1 displayed a prominent T>A motif, consistent with the different causes of base alterations in nuclear compared with mt DNA. Mutational signatures varied between patients. Changes in the principal signatures of the circulating variants over the course of the patients were not informative of disease status, possibly because of the same reasons that confounded the diagnostic utility of the circulating variants underlying the signatures. Some of the notions put forward above are hypothetical and require consecutive biopsies of tumors and organs to be verified.

A fraction of tumor‐associated mt and nuclear single nucleotide variants found in NB by targeted sequencing could be detected in the plasma of a minority of patients and were informative of disease status. Thus, both mt and nuclear variants should be regarded as potential biomarkers for liquid biopsy of NB. On the other hand, most tumor‐associated variants were not detected in the circulation. Maximizing both volume of plasma samples and depth of coverage of circulating DNA, and analyzing cerebral spinal fluid, may enhance detection of circulating variants. With regard to the latter, three of five patients in this study died of cerebral metastasis.

There are several limitations of this study that should be addressed in the future. First, the number of patients evaluated was small, precluding statistical comparison between patients. Second, patients were selected for metastatic disease, their disease relapsed or progressed, and all patients eventually died from their disease. It will be informative to study additional patients that are in long‐term remission or that were of low risk. Third, adding more genes to be sequenced may increase the number of informative circulating variants and their signatures. Furthermore, repeat biopsies of tumors and, if feasible, biopsies of multiple organs should address the challenges of tumor‐evolution and nontumor somatic mt variants, respectively. However, repeat surgical biopsies and autopsies of deceased children with NB are not yet clinical standard. Finally, quantifying the volume of NB bone marrow metastases in MIBG scans warrants further studies. Taken together, these limitations highlight the technical challenges of liquid biopsy in NB.

## AUTHOR CONTRIBUTIONS

**Lara Riehl:** Formal analysis (equal); investigation (lead); writing – original draft (equal); writing – review and editing (equal). **Medhanie Mulaw:** Conceptualization (equal); formal analysis (equal); investigation (equal); methodology (equal); writing – original draft (equal); writing – review and editing (equal). **Katharina Kneer:** Investigation (equal); methodology (equal); writing – original draft (equal). **Meinhard Beer:** Supervision (supporting). **Ambros Beer:** Supervision (supporting). **Thomas F. Barth:** Investigation (supporting); writing – original draft (supporting). **Vladimir Benes:** Investigation (supporting). **Johannes Schulte:** Resources (supporting); validation (supporting); writing – original draft (supporting). **Matthias Fischer:** Validation (supporting); writing – original draft (supporting); writing – review and editing (supporting). **Klaus‐Michael Debatin:** Supervision (supporting); validation (supporting). **Christian Beltinger:** Conceptualization (lead); funding acquisition (lead); supervision (lead); writing – original draft (lead); writing – review and editing (lead).

## CONFLICT OF INTEREST

The authors declare that they have no competing interests.

## ETHICS STATEMENT

The study was approved by the ethics committee of the University of Ulm (protocol code 25/14) and written informed consent was obtained.

## Supporting information


**FIGURE S1 No tumor‐associated circulating variants in a patient with lethal cerebral metastasis**.

**(A) Clinical course**. Chemo, chemotherapy; ASCT, autologous stem cell transplantation; cis‐RA, cis‐retinoic acid differentiation therapy; PR, partial remission; PROG; progression; D, death; MIBG trend, response assessment by MIBG; BM, bone marrow samples (filled circle: positive for NB cells); Plasma samples, time of plasma sampling; SUR, surgery and biopsy; VMA, vanillylmandelic acid; HVA, homovanillic acid.
**(B) High number of mt and nuclear somatic variants with low allelic frequency in tumor and consecutive plasma samples**. T, tumor; P1–5, consecutive plasma samples.
**(C) Mt and nuclear principal signatures differ**. Mt and nuclear principal signatures S1, S2, and S3 are shown.
**(D) Circulating mt and nuclear principal signatures do not reflect the clinical course**. Unsupervised hierarchical clustering of the principal signatures S1, S2, and S3 by the consecutive samples T (tumor) and P1–5 (plasma samples) are depicted

**TABLE S1** Patient characteristics

**TABLE S2** Nuclear genes sequenced

**TABLE S3** Chromosomal location of regions for structural analyses included in the custom NB panel

**TABLE S4** Mitochondrial and nuclear somatic variants are found in tumors (T) and consecutive plasma samples (P1–P5). Allelic frequencies are shown as %

## Data Availability

Data are available from the corresponding author upon reasonable request.
