# Supplementary figures and images for: Targeted parallel DNA sequencing detects circulating tumor‐associated variants of the mitochondrial and nuclear genomes in patients with neuroblastoma
Source: Cancer Rep (Hoboken). 2022 Jul 28;6(1):e1687. doi: 10.1002/cnr2.1687 (PMC9875664; doi:10.1002/cnr2.1687)

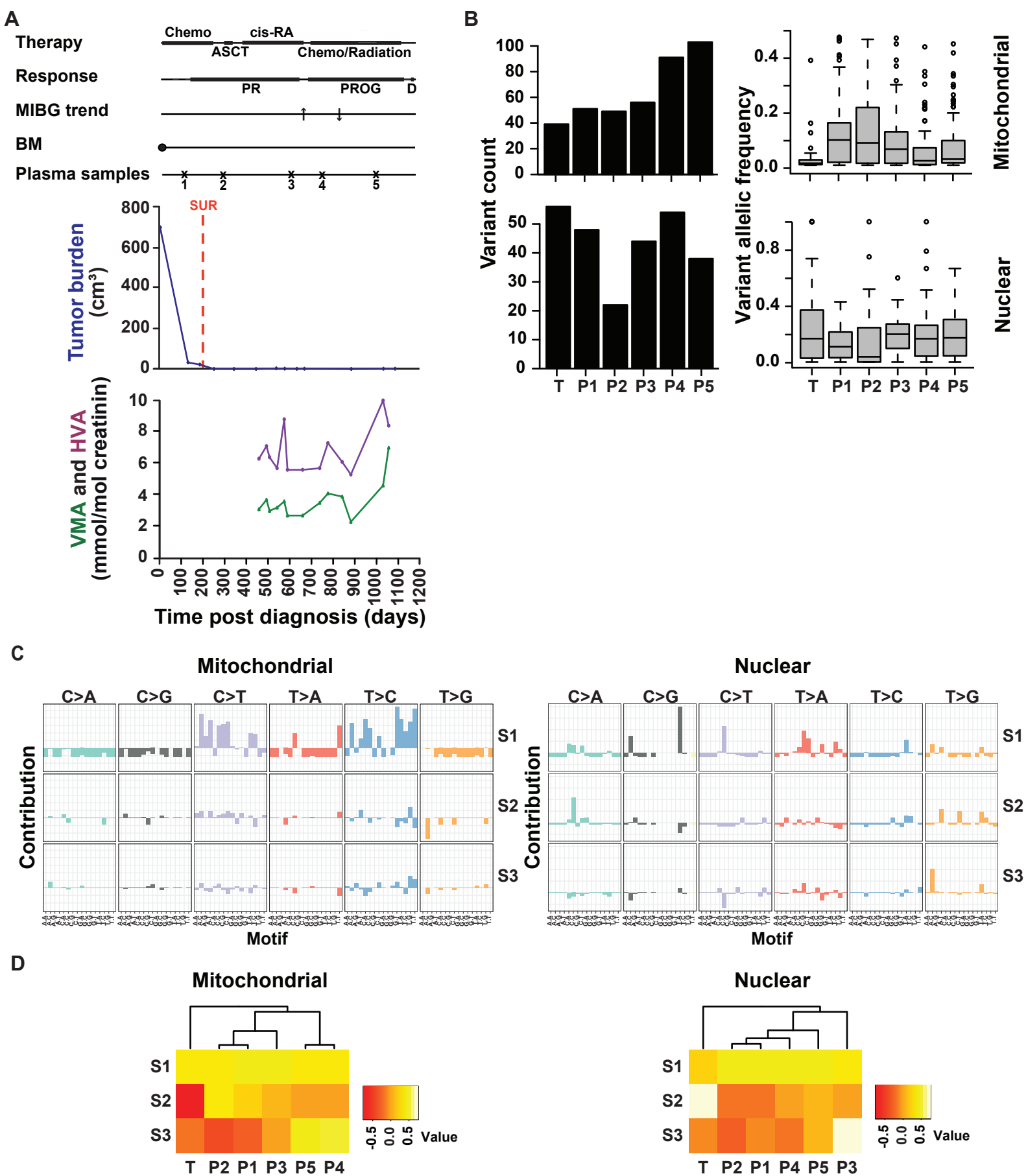

Supplemental Figure S1

Supplement: Supplementary file 1 — FIGURE S1 No tumor‐associated circulating variants in a patient with lethal cerebral metastasis. [file CNR2-6-e1687-s001.pdf]
