# Supplementary material for: Targeted parallel DNA sequencing detects circulating tumor‐associated variants of the mitochondrial and nuclear genomes in patients with neuroblastoma
Source: Cancer Rep (Hoboken). 2022 Jul 28;6(1):e1687. doi: 10.1002/cnr2.1687 (PMC9875664; doi:10.1002/cnr2.1687)
Supplement: Supplementary file 2 — (A) Clinical course. Chemo, chemotherapy; ASCT, autologous stem cell transplantation; cis‐RA, cis‐retinoic acid differentiation therapy; PR, partial remission; PROG; progression; D, death; MIBG trend, response assessment by MIBG; BM, bone marrow samples (filled circle: positive for NB cells); Plasma samples, time of plasma sampling; SUR, surgery and biopsy; VMA, vanillylmandelic acid; HVA, homovanillic acid. (B) High number of mt and nuclear somatic variants with low allelic frequency in tumor and consecutive plasma samples. T, tumor; P1–5, consecutive plasma samples. (C) Mt and nuclear principal signatures differ. Mt and nuclear principal signatures S1, S2, and S3 are shown. (D) Circulating mt and nuclear principal signatures do not reflect the clinical course. Unsupervised hierarchical clustering of the principal signatures S1, S2, and S3 by the consecutive samples T (tumor) and P1–5 (plasma samples) are depicted [file CNR2-6-e1687-s003.docx]

**SUPPLEMENTAL FIGURE S1 No tumor-associated circulating variants in a patient with lethal cerebral metastasis.**

**(A) Clinical course.** Chemo, chemotherapy; ASCT, autologous stem cell transplantation; cis-RA, cis-retinoic acid differentiation therapy; PR, partial remission; PROG; progression; D, death; MIBG trend, response assessment by MIBG; BM, bone marrow samples (filled circle: positive for NB cells); Plasma samples, time of plasma sampling; SUR, surgery and biopsy; VMA, vanillylmandelic acid; HVA, homovanillic acid.

**(B) High number of mt and nuclear somatic variants with low allelic frequency. in tumor and consecutive plasma samples.** T, tumor; P1-5, consecutive plasma samples.

**(C) Mt and nuclear principal signatures differ.** Mt and nuclear principal signatures S1, S2 and S3 are shown.

**(D) Circulating mt and nuclear principal signatures do not reflect the clinical course.** Unsupervised hierarchical clustering of the prinicipal signatures S1, S2 and S3 by the consecutive samples T (tumor) and P1-5 (plasma samples) are depicted**.**
