# Supplementary material for: Targeted parallel DNA sequencing detects circulating tumor‐associated variants of the mitochondrial and nuclear genomes in patients with neuroblastoma
Source: Cancer Rep (Hoboken). 2022 Jul 28;6(1):e1687. doi: 10.1002/cnr2.1687 (PMC9875664; doi:10.1002/cnr2.1687)
Supplement: Supplementary file 3 — TABLE S1 Patient characteristics [file CNR2-6-e1687-s005.docx]

SUPPLEMENTAL TABLE S1. Patient characteristics

| **Patient** | **Sex** | **Age at diagnosis^a^** | **Stage^b^** | **Histology^c^** | ***MYCN*^d^** | **1p del^e^** | **Risk group^f^** | **Metastasis^g^** | **Relapse/ Progress** |
| --- | --- | --- | --- | --- | --- | --- | --- | --- | --- |
| A | f | 1 | IV | 1 | + | nd | HR | 3,4 | + |
| B | m | 1 | IV | 2 | - | - | HR | 1,2,3 | + |
| C | f | 1 | IV | 1 | + | + | HR | 2,3,4 | + |
| D | m | 0 | IV | 1 | + | + | HR | 2 | + |
| E | f | 1 | IV | 4 | - | - | HR | 1,2,4 | + |

^a^ Age at diagnosis: 1, ≥18 months; 0, <18 months.

^b^ Stage of disease according to the International Neuroblastoma Staging System.^11^

^c^ Histology based on the International Neuroblastoma Pathology Classification (INPC); 1: NB

undifferentiated; 2: NB poorly differentiated; 3: NB differentiating; 4: NB, differentiation not defined.

^d^ +, amplified; -, not amplified; nd, not determined.

^e^ +, deleted; -, not deleted; nd, not determined.

^f^ HR, high-risk group according to GPOH.

^g^ Metastasis; 1: bone marrow; 2: bone; 3: distant lymph node; 4: CNS, liver, lung or skin.
