# Supplementary material for: Targeted parallel DNA sequencing detects circulating tumor‐associated variants of the mitochondrial and nuclear genomes in patients with neuroblastoma
Source: Cancer Rep (Hoboken). 2022 Jul 28;6(1):e1687. doi: 10.1002/cnr2.1687 (PMC9875664; doi:10.1002/cnr2.1687)
Supplement: Supplementary file 4 — TABLE S2 Nuclear genes sequenced [file CNR2-6-e1687-s004.docx]

SUPPLEMENTAL TABLE S2. Nuclear genes sequenced

| ABCA13 | CDK4 | HERC2 | mir152 | NBPF10 | RICTOR |
| --- | --- | --- | --- | --- | --- |
| ADAMTSL3 | CDK6 | HMCN1 | mir182 | NCAM1 | ROCK2 |
| AHNAK | CDKN2A | HRAS | mir183 | NF1 | RPTOR |
| AHNAK2 | CDKN2B | IDH1 | mir186 | NOTCH1 | RYR1 |
| ALK | CHD5 | IDH2 | mir191 | NPHP1 | SDHB |
| ANK3 | CHEK2 | IGF1R | mir21 | NRAS | SLC12A6 |
| APC | CPT1C | INTS1 | mir29a | NTRK2 | SMARCA4 |
| APOB | CREBBP | JAK2 | mir301a | OR8U1 | SMARCC2 |
| ARAF | CTNNB1 | KCNJ12 | mir30e | PALB2 | SMO |
| ARID1A | DDR2 | KDM5A | mir31 | PARK7 | SORCS2 |
| ARID1B | DMXL1 | KIAA1109 | mir335 | PDE6G | SOS1 |
| ARID2 | DNAH11 | KIF1B | mir34a | PDGFRA | SPTA1 |
| ATM | DNAH14 | KIT | mir34b | PDGFRB | SYNE2 |
| ATP10B | DNAH5 | KRAS | mir34c | PHOX2B | TCHH |
| ATR | DOCK8 | let7a2 | mir425 | PIK3CA | TERT |
| ATRX | EGFR | let7a3 | mir454 | PIK3CB | TNC |
| BARD1 | ERBB2 | LIN28b | mir633 | PINK1 | TNXB |
| BCOR | EZH2 | LRP1B | MKI67 | PLXNA3 | TP53 |
| BIRC5 | FANCM | MAP2K1 | MLL3 | PLXND1 | TRIO |
| BRAF | FAT2 | MAP2K2 | mTOR | PTCH1 | TSC1 |
| BRCA1 | FBN2 | MAP3K12 | MUC16 | PTEN | USP6 |
| BRCA2 | FBXW7 | MDM2 | MUC17 | PTPN11 | VAMP3 |
| BRD7 | FGFR1 | MDM4 | MUC5B | PTPN14 | VANGL1 |
| C3 | FLG | MET | MYC | PTPRD | VCAN |
| CADM2 | FRAS1 | mir100 | MYCL1 | RASD1 | YAP1 |
| CCER1 | FREM2 | mir10a | MYCN | RBBP6 | ZNF17 |
| CCND1 | HERC1 | mir139 | MYO9A | RBP7 |  |

Grey shading: genes implicated in the literature; red shading: genes and miRNA implicated in the TARGET database; blue shading: additional genes not implicated in NB so far but deemed to be potentially relevant based on their function.
