# Supplementary material for: Targeted parallel DNA sequencing detects circulating tumor‐associated variants of the mitochondrial and nuclear genomes in patients with neuroblastoma
Source: Cancer Rep (Hoboken). 2022 Jul 28;6(1):e1687. doi: 10.1002/cnr2.1687 (PMC9875664; doi:10.1002/cnr2.1687)
Supplement: Supplementary file 5 — TABLE S3 Chromosomal location of regions for structural analyses included in the custom NB panel [file CNR2-6-e1687-s002.docx]

SUPPLEMENTAL TABLE S3. Chromosomal location of regions for structural analyses included in the custom NB panel

| chr1:934439-935353 | chr11:72604282-72604404 |
| --- | --- |
| chr1:1347070-1347267 | chr11:76813886-76814377 |
| chr1:3225994-3226159 | chr11:81597206-81597328 |
| chr1:5816748-5816909 | chr11:86656717-86666440 |
| chr1:5837603-5837713 | chr11:91627874-91628069 |
| chr1:6098700-6098810 | chr11:95910992-95911156 |
| chr1:6118944-6119309 | chr11:110979632-110979799 |
| chr1:6738372-6738532 | chr11:113821233-113821492 |
| chr1:7617803-7618507 | chr11:114539579-114539742 |
| chr1:9034795-9035146 | chr11:116667219-116667444 |
| chr1:28562695-28564489 | chr11:117843627-117843737 |
| chr1:45976707-45988562 | chr11:118618472-118618890 |
| chr1:65300001-65300315 | chr11:118965673-118966104 |
| chr1:82733819-82734101 | chr11:119367625-119367735 |
| chr1:101185196-101204601 | chr11:123626384-123627020 |
| chr1:109472420-109472552 | chr11:125131254-125131490 |
| chr1:115880000-115900000 | chr11:127264106-127264241 |
| chr3:32994915-32995997 | chr11:131965792-131965905 |
| chr3:36414609-36415028 | chr17:19186433-19237567 |
| chr3:39449882-39450030 | chr17:19314491-19320589 |
| chr3:43732455-43732864 | chr17:26377536-26377707 |
| chr3:45998139-45998468 | chr17:32683471-32685629 |
| chr3:46414394-46415452 | chr17:35379366-35379525 |
| chr3:47576225-47576335 | chr17:37885409-37886788 |
| chr3:49113717-49113827 | chr17:41384362-41384762 |
| chr3:52084831-52090382 | chr17:46674583-46674722 |
| chr3:57232225-57233946 | chr17:51900671-51902553 |
| chr3:60107362-60107549 | chr17:54965271-54965597 |
| chr3:62355758-62358543 | chr17:58399327-58399458 |
| chr3:66024386-66024536 | chr17:62473940-62493086 |
| chr3:69775963-69776112 | chr17:67139699-67140234 |
| chr3:73111233-73112051 | chr17:68500909-68501246 |
| chr3:77366604-77366754 | chr17:69343755-69344122 |
| chr3:79936000-79956000 | chr17:70672047-70672365 |
| chr5:1210000-1255000 | chr17:72561312-72561879 |
| chr5:1295000-1345000 | chr17:74732243-74733242 |
| chr11:65467579-65467770 | chr17:78175266-78175555 |
| chr11:69390000-69410000 |  |
