# Supplementary material for: Targeted parallel DNA sequencing detects circulating tumor‐associated variants of the mitochondrial and nuclear genomes in patients with neuroblastoma
Source: Cancer Rep (Hoboken). 2022 Jul 28;6(1):e1687. doi: 10.1002/cnr2.1687 (PMC9875664; doi:10.1002/cnr2.1687)
Supplement: Supplementary file 6 — TABLE S4 Mitochondrial and nuclear somatic variants are found in tumors (T) and consecutive plasma samples (P1–P5). Allelic frequencies are shown as % [file CNR2-6-e1687-s006.pdf]

**SUPPLEMENTAL TABLE S4. Mitochondrial and nuclear somatic variants are found in tumors (T) and consecutive plasma samples (P1-P5).** Allelic frequencies are shown as %.

| | Patient | A | | | | | B | | | | | C | | | | | D | | | | | E | | | | | |
|---|---|---|---|---|---|---|---|---|---|---|---|---|---|---|---|---|---|---|---|---|---|---|---|---|---|---|---|
| **Gene** | **Variant Sample** | **T** | **P1** | **P2** | **P3** | **P4** | **T** | **P1** | **P2** | **P3** | **P4** | **T** | **P1** | **P2** | **P3** | **P4** | **T** | **P1** | **P2** | **P3** | **P4** | **T** | **P1** | **P2** | **P3** | **P4** | **P5** |
| ATP6 | chrM:9182:G:A | | | | | | | | | | | | | | | | | 15.7 | 79.0 | 12.5 | | | | | | | |
| COX1 | chrM:6045:C:T | | | | | | | | | | | | | | | | | 33.3 | 69.6 | 3.5 | | | | | | | |
| COX1 | chrM:COX1:6045:C:T | | | | | | | | 3.4 | | | | | | | | | 33.3 | 69.4 | 3.5 | | | | | | | |
| COX3 | chrM:9738:G:A/T | | | | | | | | | | | | | | | | | 14.6 | 1.2 | | | | | | | | |
| CYTB | chrM:14766:T:C | | | | | | | | | | | | | | | | | | | | | | | | | 2.3 | 9.6 |
| D-Loop | chrM:16498:G:A | | | | | | | | | 36.4 | 57.1 | | | | | | | | | | | | | | | | |
| D-Loop | chrM:16270:C:T | | | | | | | | | | | | 1.0 | | | | | 10.5 | 1.9 | | 16.9 | | | | | | 2.7 |
| D-Loop | chrM:200:A:G | | | | | | | | 5.8 | | | | | 2.3 | | 2.5 | | | | | | | | | 2.7 | 3.3 | 4.6 |
| D-Loop | chrM:217:T:C | | | | | | | | 13.5 | | | | | | | | | | | | | | | | 2.6 | 6.0 | 2.0 |
| ND1 | chrM:3720:A:G | | | | | | | | | | | | | | | | | | | | | | | | | 1.2 | 3.3 |
| ND2 | chrM:4917:A:G | | | | | | | | | | | | | | | | | | | | 27.8 | | | | | | |
| ND2 | chrM:5390:A:G | | | | | | | | | | | | | 1.2 | | | | 12.4 | 81.1 | 11.8 | | | | | | | |
| ND2 | chrM:5426:T:C | | | | | | | | | | | | | | | | | 9.6 | 75.9 | 13.2 | 16.0 | | | | | | 1.1 |
| ND4 | chrM:11467:A:G | | | | | | | | | | | | | 1.1 | | | | | | | | | | | | | |
| ND4 | chrM:11981:C:T | | | | | | | | 30.3 | | | | | | | | | 22.6 | 91.2 | 30.4 | 17.1 | | | | | | |
| ND4L | chrM::10550:A:G | | | | | | | | | | | | | | | | | | | | | | | | | | 1.1 |
| ND5 | chrM:12769:G:A | 16.1 | | | 2.7 | 48.7 | | | | | | | | | | | | | | | | | | | | | |
| ND5 | chrM:13734:T:C | | | | | | | | 26.7 | | | | | | | | | 29.9 | 74.0 | 3.3 | 7.7 | | | | | | |
| RNR2 | chrM:2706:G:A | | | | | | | | | | | | | | | | | | | | | | | | | 1.5 | 2.7 |
| TRNR | chrM:10463:T:C | | | | | | | | | | | | | | | | | | | | 33.3 | | | | | | |
| TRNT | chrM:15907:A:G | | | | | | | | | | | | | | | | | 31.3 | 84.3 | 14.7 | | | | | | | |
| TRNT | chrM:15907:A:G | | | | | | | | 47.8 | | | | | 1.4 | | | | 31.3 | 84.3 | 14.7 | | | | | | | |
| BRD7 | chr16:50354745:T:TTG | | | | | | | | | | | | | | | | | | 2.1 | | 2.1 | | | | | | |
| DOCK8 | chr9:463596:T:TTGAGCTACTG | | | | | | | | | | | 2.1 | | 100.0 | | | | | | | | | | | | | |
| IGF1R | chr15:99491968:C:CTG | | | | | | | | | | | | | | | | | | 2.1 | | 2.1 | | | | | | |
